# Cases of Neuralgia

**Published:** 1824-11

**Authors:** 

**Affiliations:** Liverpool.


ART. IV.—*Cases of Neuralgia.* Communicated by Dr. VANDEBURGH, of Liverpool.

MR. W. P., ætatis thirty, consulted me on the 24th of January, 1824, labouring under severe attacks of neuralgia. He stated having suffered from the complaint upwards of two years; that he had tried an endless variety of medicines; had applied both leeches and blisters, and still continued taking the pulvis valerianiæ in large doses, without the most remote relief.

The right side of the face was the part affected; the paroxysms were very acute, and their attacks occurred about noon. On retiring at night, owing to the distracting pains, he was frequently under the necessity of walking about his bed-room nearly throughout the night. The sight of the right eye was considerably affected during the spasm, and there was an almost constant discharge of mucus from the nostril of the same side. Upon repeated examination, the teeth appeared perfectly sound; and, as his avocations often obliged him to go to and from Manchester, his sufferings were rendered more severe.

I ordered twenty ounces of blood to be abstracted by cupping from the back of the neck, and prescribed the following purgative:—℞. Magnesiæ Sulphat. ℥iss. Infusi Rosæ, Infusi Sennæ, āā ℥iijss. fiat Mistura. Bibat cyath. vinosum tertiâ quâque horâ.

On the 25th, although the medicine had acted very freely, the complaint continued as yesterday; I therefore commenced giving two scruples of the ferri subcarbonas, four times a-day, in some preserved fruit, and desired the patient to abstain from malt and spirituous liquors.

On the 27th, he imagined the pain more severe than ever, and attributed it to the medicine. He sent for me in the evening; and, under the impression that the iron was operating to his future advantage, I urged him to continue the same.

On the 29th, great amelioration had taken place: the paroxysms were less severe, and his appetite (previously much impaired) considerably improved.

On the 30th, my patient slept well, and experienced greater animal spirits than he had done for a long time: the spasms had been, comparatively, but trifling. I increased the dose of ferri subcarbonas to fifty grains four times a-day.

On the 1st of February, he felt but little or no pain; had resumed his accustomed diet and wine, and went to Manchester. He returned on the 4th; had suffered no attacks during his journey, and felt perfectly restored. He continued the iron for some time afterward, with the addition of a small quantity of the pulv. rhubarb, to keep the primæ viæ regular.

I met him a few days since, on his return from the continent, and he has experienced no relapse of his truly distressing and most painful complaint.

Mr. E. S., ætatis twenty-nine, (November 22d, 1823,) stated that he had suffered occasionally from the most exquisite pain on the left side of the face, but more especially over the orbit of the left eye, which very considerably affected his sight. The spasms attacked him suddenly, and with such severity, that for the time he was compelled to relinquish his occupation. The pain was at one time attributed to a carious tooth, for which anodynes, accompanied with copious bleedings, had been prescribed, and ultimately the tooth was extracted; but no remedy proved availing to remove the torture which my patient endured. The paroxysms were generally aggravated at night.

I began to prescribe the ferri subcarbonas, in doses of half a drachm three times a-day, mixed in some conserve, and increased the quantity to two scruples. On the 5th of December, the complaint was entirely removed; since which time, however, he has experienced a very slight attack, but which was immediately banished by having recourse to the same remedy.

I beg leave to mention, that the first time I prescribed the subcarbonate of iron, was for a lady from Edinburgh, in December 1822, who had suffered, for a considerable period, most severely from tic douloureux. I prescribed the medicine in doses of twenty grains three times a-day; and she left Liverpool in a very improved state, promising to acquaint me with the progress of her cure; but I have not heard of her since.

I hope you will allow me the present opportunity of thanking Mr. Hutchinson for the publicity he has already given to the world, of his very successful mode of treating the various modifications of neuralgia; and also for his liberal promise of communicating to his brethren the results of his future experience on this interesting subject.

*Liverpool; the 20th Sept.* 1824.

---

ART. V.—*Observations on* Mr. EARLE's *Fracture-Bed, &c.* By E. HARROLD, Esq. Surgeon.

